# Data of radiation damage on selenomethionine-substituted single-domain substrate-binding protein

**DOI:** 10.1016/j.dib.2024.110114

**Published:** 2024-01-30

**Authors:** Ki Hyun Nam

**Affiliations:** College of General Education, Kookmin University, Seoul 02707, South Korea

**Keywords:** Selenomethionine, Radiation damage, Substrate binding protein, Crystal structure, X-ray crystallography

## Abstract

Radiation damage is an inherent issue in X-ray crystallography. It not only damages macromolecular crystals, which lowers the quality of the diffraction intensity, but results in inaccurate structural information. Among the various types of radiation damage, little is known regarding the damage to selenomethionine, an amino acid contained in some proteins. Recently, radiation damage to the selenomethionine-substituted single-domain substrate-binding domain from *Rhodothermus marinus* (SeMet-RmSBP) was investigated. Global and specific radiation damage from four datasets collected by repeatedly exposing a single RmSBP-SeMet crystal to X-rays were analyzed. The results indicated that the B-factor value of the selenium atom in selenomethionine was significantly increased compared with other atoms. To date, no images of radiation damage have been reported for selenomethionine-substituted proteins. Therefore, these data may be used to study radiation damage in macromolecular crystallography. This study provides insight into radiation damage associated with selenomethionine.

Specifications Table

**Table:** 

Subject	Biological sciences
Specific subject area	Structural Biology
Data format	Raw, Analyzed
Type of data	X-ray diffraction data, Table, Image, Graph, Figure
Data collection	Synchrotron: Pohang Light Source II (PLS-II) Beamline: 7A X-ray energy: 12660 eV Photon flux: ∼ 1 × 10^12^ photons/s Beam size: 100 × 100 µm (full width at half maximum, vertical x horizontal) Detector: ADSC Q270 CCD detector Data acquisition: 1 ° rotation / 1 s Data collection temperature: 100K
Data source location	Institution: Kookmin University City/Town/Region: Seoul Country: Republic of Korea
Data accessibility	1. Raw data diffraction images Repository name: ZENODO Diffraction data for SeMet-RmSBP (Radiation damage study) - Digital Object Identifier: https://doi.org/10.5281/zenodo.10023763 - Direct URL to data: https://zenodo.org/records/10023763 2. Structure factor and coordinate Repository name: Protein Data Bank (PDB, http://rcsb.org) (1) Data I: X-ray dose I - PDB code: 8WXM - Data identification number: https://doi.org/10.2210/pdb8WXM/pdb - Direct URL to data: https://www.rcsb.org/structure/8WXM (2) Data 2: X-ray dose III - PDB code: 8WXN - Data identification number: https://doi.org/10.2210/pdb8WXN/pdb - Direct URL to data: https://www.rcsb.org/structure/8WXN (3) Data 3: X-ray dose III - PDB code: 8WXO - Data identification number: https://doi.org/10.2210/pdb8WXO/pdb - Direct URL to data: https://www.rcsb.org/structure/8WXO (4) Data 4: X-ray dose IV - PDB code: 8WXP - Data identification number: https://doi.org/10.2210/pdb8WXP/pdb - Direct URL to data: https://www.rcsb.org/structure/8WXP
Related research article	K.H. Nam, Radiation Damage on Selenomethionine-Substituted Single-Domain Substrate-Binding Protein, Crystals (2023) [1]https://doi.org/10.3390/cryst13121620

## 1 Value of the Data

• X-ray dose-dependent diffraction data for radiation damage to SeMet-RmSBP were collected.
• The diffraction data damaged by radiation for SeMet are reported.
• The global and specific radiation damage of SeMet-RmSBP were analyzed.
• The temperature factors of the SeMet residue and its selenium atom in RmSBP were analyzed.

## 2 Background

Radiation damage is an inherent experimental problem in macromolecular crystallography (MX) [2]. The global and specific radiation damage can occur in crystals exposed to X-rays [3], [4], [5]. This reduces the diffraction intensity of the macromolecular crystal, thus preventing the collection of high-resolution diffraction data or the acquisition of complete three-dimensional structural diffraction data [3,4]. In addition, diffraction data containing radiation damage may contain electron density maps that are biologically irrelevant [6], [7], [8]. To minimize radiation damage during X-ray crystallography, it is important not only to reduce X-ray exposure time, but also to understand the radiation damage phenomenon to establish a data collection strategy. Selenomethionine (SeMet) is a constituent amino acid in proteins [9]. Because it contains a high atomic mass of selenium (Se), it is widely used for single-wavelength anomalous diffraction (SAD) or multi-wavelength anomalous diffraction (MAD) phasing techniques in protein crystallography [10,11]; however, the radiation damage phenomenon is not well understood. Typical SBP proteins recognize a substrate using two α/β domains [12], whereas the single-domain substrate-binding protein from *Rhodothermus marinus* (RmSBP) has a single α/β domain. The crystal structure and flexibility of RmSBP have been established [12], [13], [14], but its molecular function is unknown. To better understand this process, radiation damage to selenomethionine-substituted RmSBP (SeMet-RmSBP) was used as a model sample, rather than focusing on the identification of biological function [1].

## 3 Data Description

**Fig. 1:**
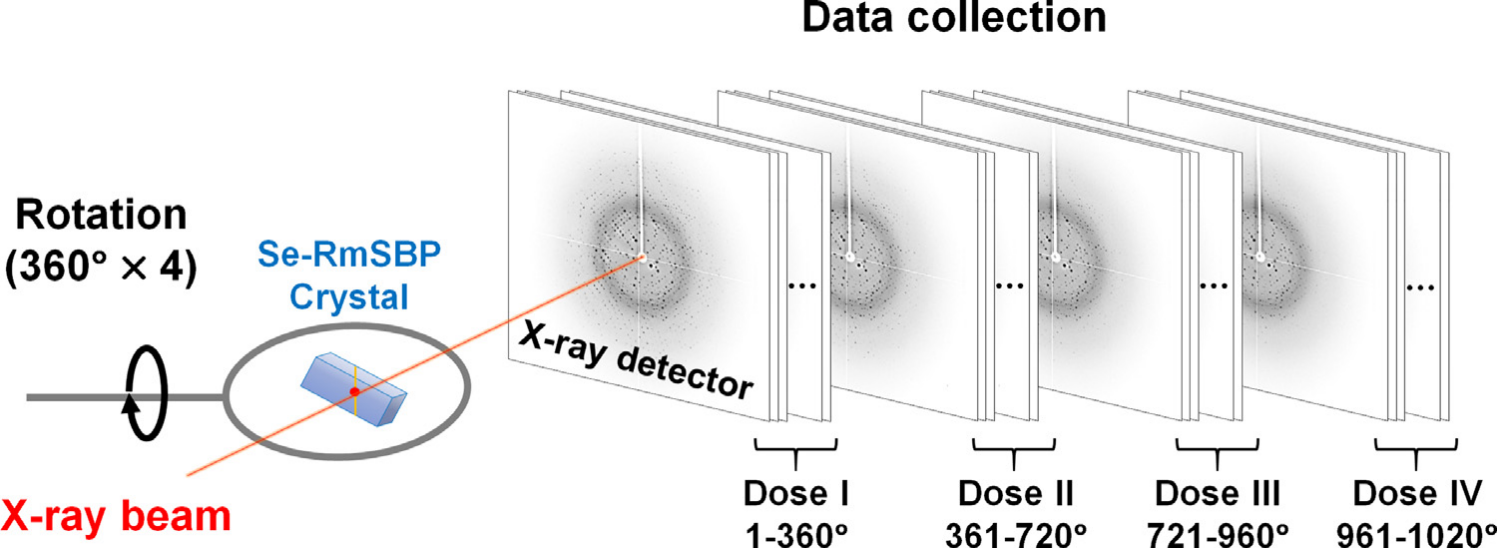
Data collection procedure for the radiation damage analysis of SeMet-RmSBP.

SeMet-RmSBP was overexpressed in *Escherichia coli* by replacing methionine with selenomethionine during protein expression. SeMet-RmSBP crystals were grown using a hanging drop vapor diffusion method with a crystallization solution containing 0.1 M Na-acetate (pH 4.5), 0.2 M MgCl_2_, and 10% (w/v) PEG 3350. After soaking the single crystal in a cryoprotectant solution, it was mounted on a goniometer at 100 K. The crystal was then rotated and exposed to X-rays to collect diffraction data. Changes in radiation damage to the SeMet residue and its selenium atom were observed based on the cumulative X-ray exposure. When multiple crystal samples were used, the quality of each crystal was not completely identical with respect to shape, size, and quality. Therefore, the diffraction intensity and radiation damage were not directly comparable, and the diffraction data were collected from a single crystal by rotating it 360°. The same crystal was subjected to three additional X-ray exposures in the same manner to collect the diffraction data (Fig. 1).

**Fig. 2:**
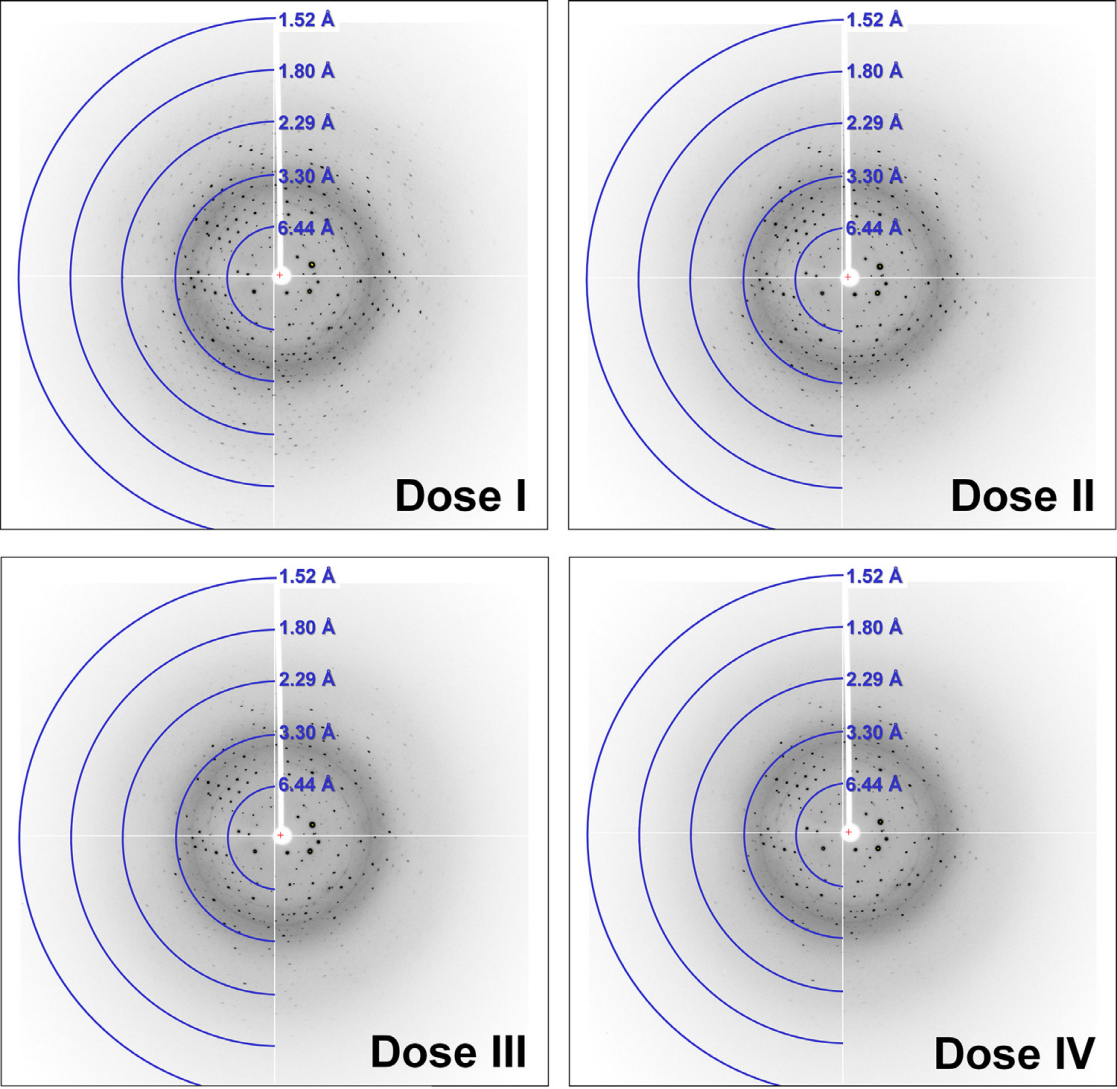
Diffraction image of SeMet-RmSBP for Dose I, II, III, and IV at oscillation angle 100°.

**Fig. 3:**
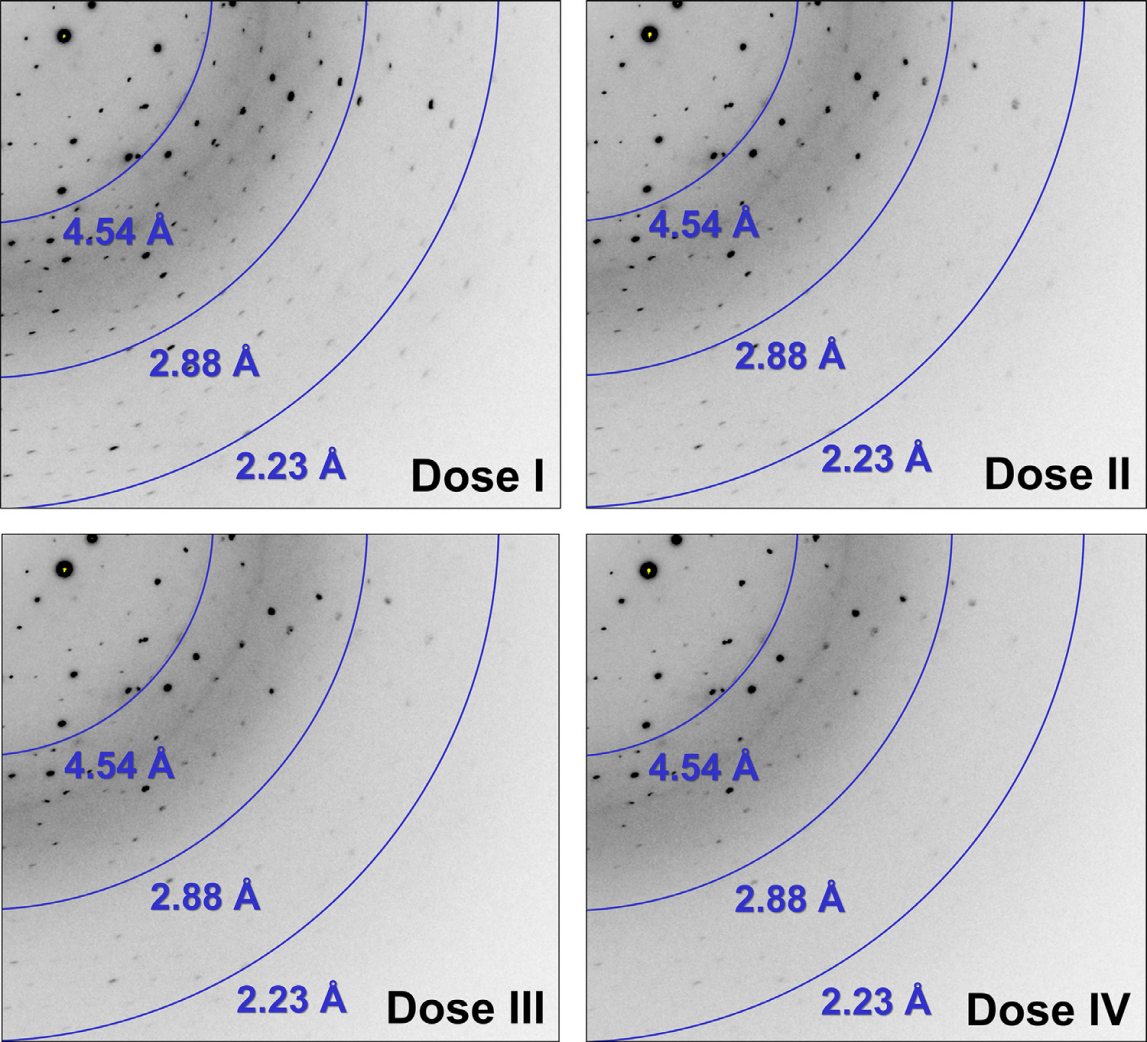
Close-up view of the diffraction image of SeMet-RmSBP for Dose I, II, III, and IV at oscillation angle 100°.

The diffraction data collected from 1, 2, 3, and 4 X-ray exposures of SeMet-RmSBP crystals were designated Dose I, II, III, and IV, respectively. For the Dose I dataset, images 1–6 were missing. The reflection number in the first and last images was low with relatively weak diffraction intensity. These were likely caused by the complete synchronization of the data collection instrument and devices (e.g., shutter, detector, data server) on the beamline. Accordingly, the number of images for Dose I deposited in ZENODO is 354, and the number of images for Dose II, III, and IV was 360. As the number of X-ray exposures increased, the intensity and number of Bragg peaks observed in the image decreased (Figs. 2 and 3).

In a previous study, data processing was performed on images 30–330 to exclude the first and last image areas; however, the resolution of the datasets may be enhanced by including additional images from 1–30 and 331–360 that were not processed. Here, the results of all of the images were processed with the aim of using the deposited data. The datasets were automatically analyzed using the Xia2 program to avoid data processing bias. Doses I, II, III, and IV were 1.46, 1.56, 1.63, and 1.70 Å, respectively, showing improved resolution compared with the previous report (Doses I, II, III, and IV were 1.50, 1.58, 1.68, and 1.75 Å). As previously reported, with an increase in X-ray exposure, the diffraction limit and I/sigma of SeMet-RmSBP tended to decrease, whereas Rmerge, Rmeas, Rpim, and B-factor tended to increase (Table 1).

**Table 1:** Data collection statistics.

Data collection	Dose I	Dose II	Dose III	Dose IV
X-ray source	Beamline 7A, PLS-II	Beamline 7A, PLS-II	Beamline 7A, PLS-II	Beamline 7A, PLS-II
Wavelength (Å)	0.97934	0.97934	0.97934	0.97934
Space group	C2	C2	C2	C2
Number of images	354	360	360	360
Cell dimensions (Å, °)				
a	63.64	63.69	63.57	63.54
b	63.90	64.12	64.08	64.15
c	69.63	69.72	69.61	69.58
α	90.00	90.00	90.00	90.00
β	96.04	96.07	96.13	96.10
γ	90.00	90.00	90.00	90.00
Resolution (Å)	39.07–1.46 (1.48–1.46)	39.15–1.56 (1.58–1.56)	36.51–1.63 (1.65–1.63)	36.49–1.70 (1.72–1.70)
Total observations	313240 (46917)	283449 (11998)	250095 (10892)	220021 (9473)
Unique reflections	46917 (1789)	39653 (1755)	34769 (1493)	30605 (1301)
Completeness (%)	97.2 (75.0)	99.3 (89.9)	99.1 (85.7)	99.0 (83.9)
Multiplicity	6.7 (4.5)	7.1 6.8)	7.2 (7.3)	7.2 (7.3)
<I/sigma>	7.7 (1.0)	7.1 (1.2)	6.2 (1.1)	5.9 (0.9)
Rmerge	0.114 (1.323)	0.122 (1.628)	0.152 (2.102)	0.172 (2.566)
Rmeas	0.124 (1.49)	0.132 (1.762)	0.164 (2.262)	0.186 (2.765)
Rpim	0.047 (0.691)	0.049 (0.667)	0.061 (0.830)	0.069 (1.021)
CC1/2	0.996 (0.462)	0.997 (0.675)	0.996 (0.614)	0.996 (0.738)
Wilson B-factor (Å^2^)	15.944	20.438	23.141	31.668

**Fig. 4:**
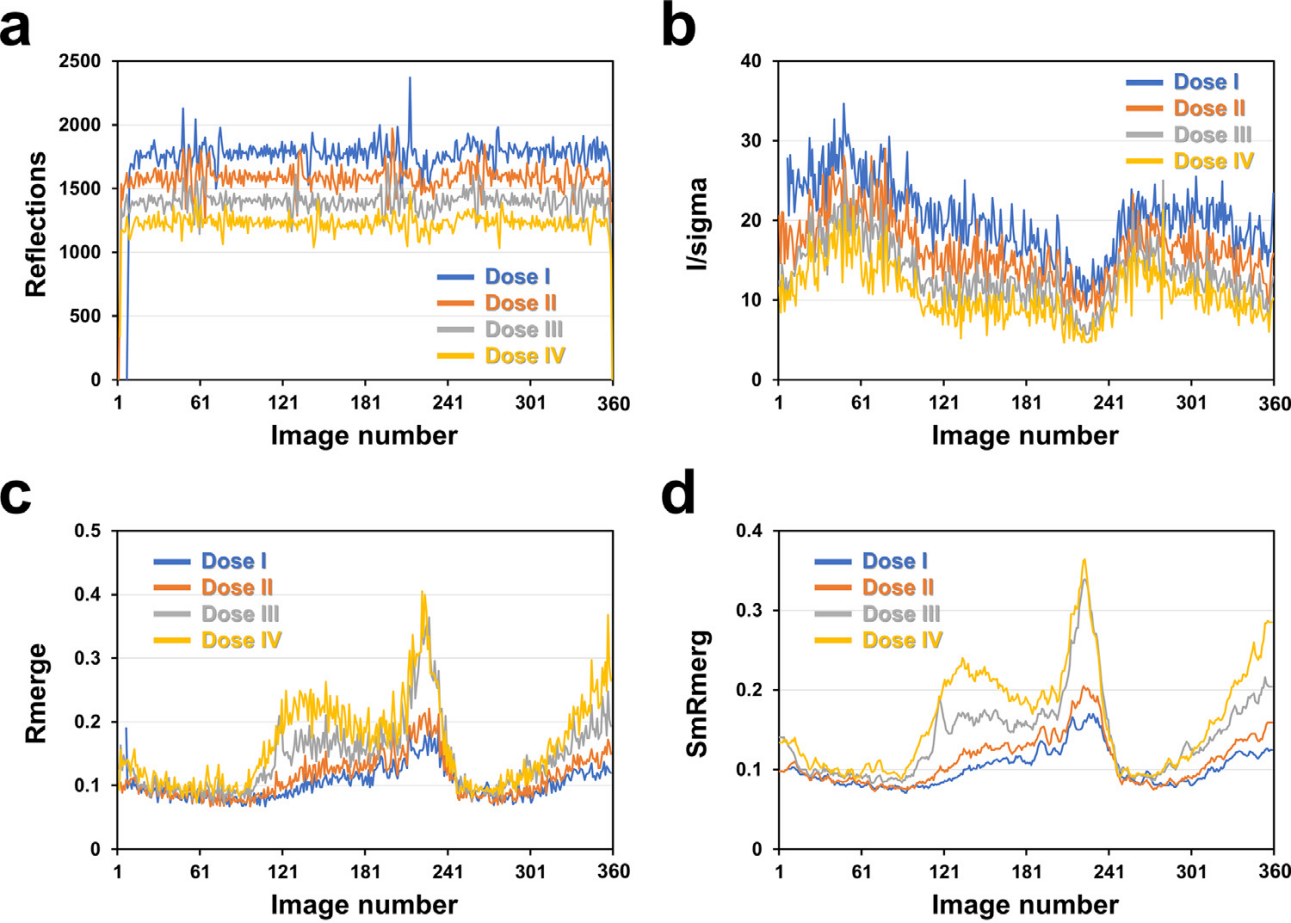
Analysis of SeMet-RmSBP for Dose I, II, III, and IV datasets. Plot of (A) number of reflections, (B) I/sigma, (C) Rmerge, and (D) SmRmerge of SeMet-RmSBP for datasets (Doses I–IV).

Next, the total reflections, I/sigma, Rmerge, and SmRmerge for each dataset covering the entire dataset are presented (Fig. 4). With an increase in X-ray exposure, the number of reflections and I/sigma decreased, whereas Rmerge and SmRmerge tended to increase. Here, I/sigma and Rmerge values did not consistently follow a trend in each dataset and varied depending on the exposure angle of the crystal to the X-rays. This variation may be attributed to differences in the location within the crystal volume where the X-rays are exposed.

As discussed earlier, because of the synchronization problem of the beamline equipment, the number of reflections was low in the initial and final images, which is evident from the profile of the reflections for Doses I, II, III, and IV (Fig. 4a). Accordingly, when reusing SeMet-RmSBP images in the future, it is appropriate to process the data by excluding early and late images.

**Fig. 5:**
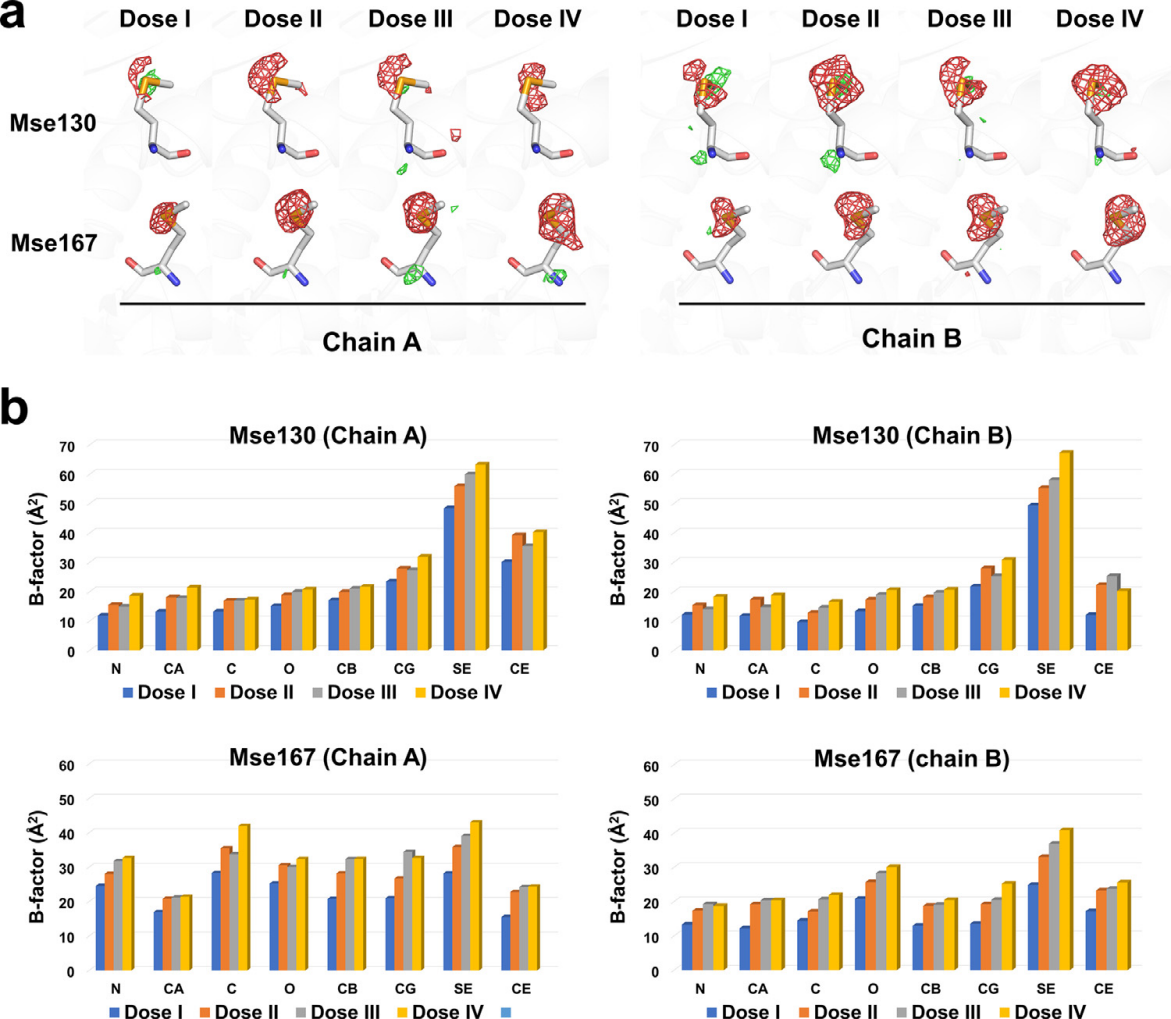
Radiation damage to the SeMet residue in SeMet-RmSBP. (A) mFo–DFc (green mesh, 3σ; red mesh, −3σ) electron density map of the Mse130 and Mse167 residues of two SeMet-RmSBP molecules in the asymmetric unit from the datasets (Doses I–IV). (B) B-factor values of the Mse130 and Mse167 residues of two SeMet-RmSBP molecules in the asymmetric unit from the datasets (Doses I–IV). The original data for the figures were obtained from a previous study [1] and have been modified.

The resolutions of Dose I, II, III, and IV of the structure factor and coordinate deposited in PDB were 1.50, 1.58, 1.68, and 1.75 Å, respectively. Meanwhile, the quality of the electron density map depended upon its resolution. Accordingly, during the analysis of the SeMet residue, the electron density map obtained by processing and refining the Dose I, II, III, and IV data were unified at a 1.7 Å resolution. As the X-ray exposure time increased, the negative electron density map at the Se atom site of SeMet concomitantly increased (Fig. 5); however, a negative density map for other atoms in SeMet was not observed. The results indicate that the Se atom is more sensitive to radiation damage compared with other atoms.

## 4 Experimental Design, Materials and Methods

The experimental procedure and detailed information for the present study have been previously reported [1]. The gene encoding RmSBP, excluding the signal peptide, was cloned into the pET28a vector and transformed into *E. coli* BL21 (DE3) cells. Recombinant SeMet-substituted RmSBP was prepared using the SeMet expression kit and protein expression was induced by the addition of 0.5 mM isopropyl β-D-1-thiogalactopyranoside. Recombinant RmSBP-SeMet was purified using Ni-NTA affinity and size exclusion chromatography. RmSBP-SeMet crystals were grown using the hanging drop vapor diffusion method at 22 °C. The RmSBP crystals were grown under a solution containing 0.1 M sodium acetate, pH 4.5, containing 0.2 M magnesium chloride and 10% (w/v) polyethylene glycol 3350. X-ray diffraction data were collected at beamline 7A at Pohang Light Source II (PLS-II, Pohang, Republic of Korea). The vertical and horizontal X-ray sizes were both 100 µm (full width at half maximum, FWHM) at sample position. The SeMet-RmSBP crystal was cryoprotected using a crystallization solution supplemented with 20% (v/v) glycerol for 5 s. The cryoprotected crystal in cryoloop was mounted on the goniometer head of a diffractometer, and then flash-cooled at 100 K under a nitrogen-gas stream. The crystal was rotated 360° in the goniometer and exposed to X-rays for 1 s per 1° rotation to collect diffraction data. Three additional sets of X-ray diffraction data were collected on the same crystal using the same method. The X-ray diffraction images were automatically indexed, integrated and scaled using the Xia2 program [15]. Electron density maps were obtained by the molecular replacement method with MOLREP (version 11.2.08) [16] in CCP4 suite using the crystal structure of the native RmSBP (PDB code 5Z6V) [12]. Manual model building was performed using the COOT (version 0.9.6) program [17]. Final structure refinement was performed using phenix.refine in PHENIX [18]. The diffraction images were deposited in ZENODO (https://zenodo.org/). The structure factor and coordinates were deposited in the Protein Data Bank (https://www.rcsb.org/).

## Limitations

Based on the electron density map, we confirmed that selenomethionine was substituted in RmSBP, but the occupancy of SeMet could not be accurately measured.

## Ethics Statement

This work meets the ethical requirements for publication in this journal. This work does not involve human subjects, animal experiments, or any data collected from social media.

## CRediT authorship contribution statement

**Ki Hyun Nam:** Data curation, Formal analysis, Validation, Visualization, Writing – original draft, Funding acquisition.

## Data Availability

Diffraction data for SeMet-RmSBP (Original data) (ZENODO). Crystal structure of Se-RmSBP (Dose I) (Original data) (PDB). Crystal structure of Se-RmSBP (Dose II) (Original data) (PDB). Crystal structure of Se-RmSBP (Dose III) (Original data) (PDB). Crystal structure of Se-RmSBP (Dose IV) (Original data) (PDB).
